# Multiple Plant Growth–Promoting Activities Exhibited by Root-Associated Bacteria Isolated From Bamboo and Corn

**DOI:** 10.1155/ijm/6374935

**Published:** 2025-03-11

**Authors:** Camille Andrea R. Flores, Maria Auxilia T. Siringan, Mary Ann Cielo V. Relucio-San Diego

**Affiliations:** Microbiological Research and Services Laboratory, Natural Sciences Research Institute, University of the Philippines, Diliman, Quezon City, Metro Manila, Philippines

**Keywords:** 16S rRNA sequencing, bacterial diversity, bamboo, corn, plant growth–promoting bacteria

## Abstract

Plant growth–promoting bacteria found in the plant roots and rhizosphere stimulate growth and reduce plant diseases through various direct and indirect mechanisms. They are proven as efficient biofertilizers that enable farmers to reduce or eliminate the use of expensive and environmentally harmful chemical fertilizers. The goal of this study was to isolate, characterize, and identify nitrogen-fixing bacteria with additional plant growth–promoting traits from the roots of bamboo (*Bambusa* sp.) and corn (*Zea mays* L.) grown in Cagayan Province, Philippines. A total of 27 bacteria were isolated and identified based on 16S rRNA gene sequencing and phylogenetic analysis. Selected isolates were also subjected to whole-genome sequencing to obtain accurate identification. The isolates were classified into 12 genera, the majority of which belonged to *Leclercia*, *Pantoea*, *Klebsiella*, and *Exiguobacterium*. Assays for four plant growth–promoting activities revealed that all isolates exhibited at least two activities in vitro. Four isolates (15%) tested positive for the nitrogen-fixation gene *nifH*, which was mostly detected in *Klebsiella* isolates. Eleven (41%) solubilized phosphate and *Pantoea* isolates showed the highest potential. All strains (100%) synthesized indole-3-acetic acid (IAA), and 24 (89%) produced siderophores. Notably, *Enterobacter roggenkampii* strain B1-01 and *Klebsiella oxytoca* strain B1-04 displayed all the examined plant growth–promoting traits. Our findings demonstrated that the roots of bamboo and corn host a variety of beneficial bacteria exhibiting significant plant growth–promoting activities under in vitro conditions. These strains could be used for future investigations into microbe–plant interactions and have the potential to be harnessed for various agricultural applications.

## 1. Introduction

To meet the global food demand, intensive farming practices rely on the continuous application of chemical fertilizers to achieve high yields. However, overuse of chemical inputs can result in soil toxicity, water pollution, and disruption of the soil–plant–microbe interactions by increasing nutrient mobilization and acquisition [1]. Another constraint for the farmers particularly in developing countries is the rising cost of fertilizers, herbicides, and pesticides. To address these challenges, the utilization of plant growth–promoting bacteria (PGPB) in agriculture emerges as a promising strategy. This group of microorganisms is known for its ability to enhance plant growth and provide protection from biotic and abiotic stresses. By harnessing the beneficial properties of PGPB, farmers can increase crop yield while simultaneously reducing the adverse environmental impacts associated with traditional agrochemicals.

PGPB consists of taxonomically unrelated bacteria found in the rhizosphere as free-living cells, attached to the root surface, or within the root tissue as endophytes [2]. PGPBs perform their functions in plant growth promotion and biocontrol through several direct and indirect mechanisms. Direct mechanisms include nutrient acquisition such as nitrogen (N) fixation; phosphate solubilization; iron sequestration by siderophores; and production of phytohormones like auxins (indole-3-acetic acid (IAA)), cytokinins, gibberellins, and ethylene modulation through the 1-aminocyclopropane-1-carboxylate (ACC) deaminase enzyme. Indirect mechanisms such as the production of antibiotics and hydrolytic enzymes, competition for nutrients and space, and induced systemic resistance may protect the plant from pathogenic bacteria, fungi, and nematodes [3]. In addition, some PGPBs alleviate the effects of abiotic stresses on the plants, such as drought, salinity, low temperature, and heavy metals [4].

Over the last two decades, researchers have successfully isolated and characterized PGPB from the rhizosphere of various plants worldwide and their beneficial effects on crop growth and yield have been well documented. In the Philippines, only a few plant species were investigated for associated beneficial soil bacteria including wild lily, sugarcane, nipa palm, rice, and cacao [5–8]. Corn (*Zea mays* L.), which belongs to the family Poaceae (formerly Gramineae), ranks second to rice as the most important staple crop in the Philippines [9]. Notably, the province of Cagayan is one of the top corn producers in the country [10]. Corn production requires high input of nutrients such as N, phosphorus (P), and potassium (K). Another member of Poaceae, bamboo, is a vital alternative crop in the country consisting of 62 species, 21 of which are endemic [11]. Because of its perennial lifestyle, bamboo is recognized for its capacity to host a greater abundance of microbes than conventional crops, which are predominantly annual monocots [12, 13]. Several studies on plant growth–promoting (PGP) microorganisms associated with corn and bamboo were conducted in other regions and have shown good results in laboratory and pot experiments [14–16]. Thus, investigating the distribution and diversity of native bacterial populations of bamboo and corn, and their potential as plant growth promoters, is essential. Furthermore, the effectiveness of PGPB as inoculants is influenced by factors such as soil, climate, and plant genotype [17], signifying the need for additional candidate PGPB strains that are effective for specific local crops or soil types.

This study is aimed at isolating bacteria from bamboo and corn roots and assess their PGP capabilities, including N fixation, phosphate solubilization, IAA synthesis, and siderophore production under in vitro conditions. Subsequently, the potential PGPB strains were identified using 16S rRNA sequencing and phylogenetic analysis or whole-genome sequencing (WGS).

## 2. Materials and Methods

### 2.1. Sample Collection and Isolation of Potential N-Fixing Bacteria

Three bamboo and five corn root samples were collected from the municipality of Barangay Magogod, Amulung, Province of Cagayan, Philippines (17°52⁣′4.81⁣^″^ N; 121°44⁣′48.54⁣^″^ E) in January 2021. The samples were placed in separate plastic bags and kept in an icebox during transport. About 10 g of roots from each sample was weighed and washed with tap water to remove soil and other debris. Root samples were then surface sterilized with 3.5% sodium hypochlorite for 5 min and then rinsed three times using sterile distilled water. Disinfected root samples were serially diluted up to 10^–3^ using 0.1% peptone water [18]. Ten-milliliter aliquots of the 10^–3^ dilution were transferred onto 100 mL of each of the four N-free basal media (i.e., NF8, NF5, NF6 with 2% glucose, and NF6 with 1% glucose) for the enrichment of N-fixing bacteria based on a discussion with Dr. Peter Jurtshuk (1988) (Supporting File S1). Enrichment cultures were incubated at room temperature (28°C) for 24–48 h. From the enrichment culture, 100 *μ*L was spread plated on Burk's N-free (BMA) agar plates (HiMedia, India) and incubated at 28°C for 24–48 h. Morphologically different colonies were purified by streaking onto fresh BMA plates. The colony morphology (size, shape, margin, color, etc.) and cellular morphology (Gram-stain reaction) of each isolate were observed. Pure cultures were stored in Burk's medium broth with 20% glycerol at −20°C and at −80°C for future use. Cultures were also freeze-dried using the Labconco FreeZone 4.5 Liter Benchtop Freeze Dry System for long-term storage.

### 2.2. Screening of N-Fixing Bacteria for Additional PGP Activities

#### 2.2.1. Detection of *nifH* Gene

Bacterial isolates that were enriched in NF8, NF5, NF6 with 2% glucose, and NF6 with 1% glucose and grown on BMA agar plates were considered putative N-fixing strains (diazotrophs). The strains were further analyzed by amplifying the gene encoding for nitrogenase reductase enzyme (*nifH*) using primers IGK3 5⁣′-GCIWTHTAYGGIAARGGIGIATHGGIAA-3⁣′ and DVV 5⁣′-ATIGCRAAICCICCRCAIACIACRTC-3⁣′ by PCR [19]. Each 20-*μ*L reaction contained 12 *μ*L GoTaq Green Master Mix (Promega Corporation, Madison, Wisconsin, United States), 1 *μ*L each of 10 *μ*M forward and reverse primers, 4 *μ*L genomic DNA, and 2 *μ*L nuclease-free water. The amplification procedure included an initial denaturation step at 94°C for 5 min, 35 cycles of denaturation at 94°C for 30 s, annealing at 53.5°C for 30 s, extension at 72°C for 30 s, and final extension at 72°C for 3 min. Nuclease-free water was used as the negative control for all amplifications. PCR products were resolved in 1% agarose gel in 1X TAE buffer stained with GelRed (Biotium). Amplicons showing the expected size of 383 bp were submitted to Macrogen (Korea) for sequencing. Consensus sequences were obtained using Molecular Evolutionary Genetics Analysis (MEGA X) software. The BLASTX program of the NCBI was used to identify the protein encoded by the nucleotide sequences.

#### 2.2.2. Phosphate Solubilization

Phosphate solubilization activity of the isolates was determined using Pikovskaya's (PVK) agar with 0.5% tricalcium phosphate as the sole P source [20]. Using an inoculating needle, the isolates were spot-inoculated at three equidistant points on the surface of PVK agar. The plates were incubated at 28°C for 5 days. The formation of a clear zone (halo) around the colony indicated a positive result. The phosphate solubilization index (PSI) which is an estimate of relative P solubilizing efficiency was computed as follows [21]:

(1) $$\begin{array}{c} \text{PSI}=\frac{\text{total diameter }\left(\text{colony}+\text{halo zone}\right)\;}{\text{colony diameter}} \end{array}$$

Bacteria were categorized into low (PSI < 2), intermediate (2 < PSI < 3), and high (PSI > 3) solubilization potential [22].

#### 2.2.3. IAA Production

Production of IAA at 72 and 144 h was determined using Salkowski's method [23, 24], as described by Fang et al. [25]. Briefly, bacterial isolates were suspended in 10-mL yeast mannitol broth (YMB) containing 1 mL of 4% Congo red per liter. The tubes were incubated at 28°C at 150 rpm for 24 h. Subsequently, 5 *μ*L of the YMB culture was inoculated into 5 mL of King's B broth supplemented with 1 mM L-tryptophan as the IAA precursor. King's B broth cultures were incubated at 28°C and 150 rpm for 72 h for one setup and at 144 h for another setup. Bacterial cells were then removed by centrifugation for 10 min at 4000 rpm. One hundred microliters (100 *μ*L) of the supernatant was mixed with 100 *μ*L of Salkowski's reagent (0.5 M FeCl_3_ 1.0 mL, concentrated H_2_SO_4_ 30 mL, and distilled water 50 mL) in a 96-well microplate. This was done in triplicate for each isolate. The mixture was then allowed to react for 30 min in the dark at room temperature. The absorbance was measured at 530 nm. IAA content was extrapolated using a standard IAA regression curve prepared from King's B medium with IAA in the range of 0–100 *μ*g/mL [26].

#### 2.2.4. Siderophore Production

Siderophore production using chrome azurol S (CAS) liquid assay was determined based on the methods described by Fang et al. [25] and Schwyn and Neilands [27]. For each isolate, triplicate 100-*μ*L aliquots of King's B culture used in the IAA assay were transferred to a 96-well microplate. For each well, 100-*μ*L CAS reagent [27] was added. Plates were incubated at room temperature for 60 min, and the absorbance was measured at 630 nm. Siderophore production was calculated using the following formula:

(2) $$\begin{array}{c} \text{Siderophore production units }\left(\text{SPU}\right)=\left(\frac{\text{Ar}-\text{As}}{\text{Ar}}\right)\times 100 \end{array}$$

where Ar is the absorbance of King's B broth, the reference solution, and As is the absorbance of the sample. Percentages of siderophore units of more than 10% were considered positive for siderophore production [28].

Finally, the PGP traits exhibited by the isolates were intersected in an UpSet plot using the Intervene online software [29] to visualize their multifunctional potential.

### 2.3. Biochemical Characterization of Selected Isolates

Pure cultures of selected isolates with promising PGP activities were also subjected to biochemical characterization using the Analytical Profile Index (API) 20E test strips (BioMerieux, France) and Biolog GEN III MicroPlate System (Biolog, Hayward, California, United States) following the manufacturers' instruction. The API 20E strip includes 20 miniature biochemical tests that are used to identify enteric and other nonfastidious, Gram-negative rods. On the other hand, the Biolog GEN III MicroPlate consists of 71 types of carbon source and 23 chemical sensitivity assay tests for the identification of Gram-negative and Gram-positive bacteria.

### 2.4. Molecular Identification

#### 2.4.1. Genomic DNA Isolation and PCR

Bacterial genomic DNA was extracted using the Isolate II Genomic DNA Kit (Meridian Bioscience, Cincinnati, Ohio, United States) according to the manufacturer's instructions. Universal primers 27F (5⁣′-AGAGTTTGATCMTGGCTCAG-3⁣′) and 1492R (5⁣′-TACGGYTACCTTGTTACGACTT-3⁣′) were used to amplify the nearly full-length sequence of the 16S rRNA gene [30] spanning approximately 1400 bp [31]. Each PCR mix contained 12.5 *μ*L GoTaq Green Master Mix (Promega Corporation, Madison, Wisconsin, United States), 0.5 *μ*L each of 10 *μ*M forward and reverse primers, 1 *μ*L genomic DNA, and nuclease-free water to bring to a total volume of 25 *μ*L. The PCR cycling conditions were as follows: initial denaturation at 94°C for 5 min, followed by 30 cycles of denaturation at 94°C for 1 min, annealing at 50°C for 45 s, extension at 72°C for 2 min, and a final extension at 72°C for 10 min. Nuclease-free water was used as the negative control for all amplifications. Amplicons were observed by agarose gel electrophoresis using 0.7% agarose in 1X TAE buffer stained with GelRed (Biotium Inc., Fremont, California, United States).

PCR products were sent to Macrogen (South Korea) for sequencing. DNA fragments of the forward and reverse primers were assembled using the MEGA X software [32]. The consensus 16S rRNA sequences were compared with the sequences of bacterial strains available in the National Center for Biotechnology Information (NCBI) GenBank database (http://www.ncbi.nlm.nih.gov) using the BLASTn program to determine their taxonomic affiliation.

#### 2.4.2. Phylogenetic Analysis

Partial 16S rRNA sequences (~1400 bp) of the closely related reference strains were downloaded from the NCBI Nucleotide database. The sequence of the archaeal species *Pyrolobus fumarii* was also downloaded to be used as an outgroup taxon for phylogenetic analyses [33]. The obtained sequences together with the sequences of the PGPB isolates were aligned using ClustalW in MEGA X to infer a possible evolutionary relationship among the bacteria. The optimal model for DNA substitution was determined for each phylogenetic tree and constructed using the maximum likelihood (ML) method (bootstrap = 1000). Another phylogenetic tree comprised solely of the 27 PGPB isolates was constructed in the same manner. The resulting tree was exported in the Newick format and uploaded to the Interactive Tree of Life (iTOL v6) [34] to include the results of PGP assays and their possible molecular identification.

#### 2.4.3. WGS Analysis

Selected isolates which were not given definitive taxonomic ranking using the 16S rRNA sequencing, BLAST, and phylogenetic analysis were subjected to WGS and pertinent bioinformatics analysis to establish their taxa. Briefly, the culture pellets were maintained in RNAlater solution and then submitted to Macrogen (South Korea) for WGS. After DNA extraction, a DNA library was constructed using the TruSeq DNA Nano sample preparation kit and then paired-end (2 × 150 bp) sequencing was performed using the Illumina HiSeq X Ten technology. The sequencing data were processed and analyzed via the Galaxy web platform (usegalaxy.org.au) [35]. FastQC version 0.11.9 [36] was used to check the quality of the FASTQ files, and Trimmomatic version 0.36 [37] was used to remove adapter sequences. De novo assembly was performed with Unicycler version 0.5.0 [38]. The completeness and quality of the genome assembly were assessed through Benchmarking Universal Single-Copy Orthologs (BUSCO) version 5.3.2 [39] and Quality Assessment Tool (QUAST) version 5.2.0 [40]. The optimal assembly was submitted to GTDB-Tk v1.7.0 [41] on KBase [42] to identify the taxonomic ranking of the isolates.

## 3. Results

### 3.1. Isolation of Putatively Diazotrophic PGPB

A total of 27 bacterial isolates from root samples of plants belonging to family *Poaceae* were isolated and purified using N-free media, consisting of nine from bamboo (Isolate Code B1-01 to B3-10) and 18 from corn (C1-11 to C5-34). Most of the isolates on BMA exhibited round colonies with an entire margin and glistening surface after 48-h incubation. Colony sizes ranged from 0.5 to 9.0 mm while some are spreading. The strains exhibited different pigmentation such as white, light yellow to dark yellow, and light orange. Majority of the isolates were Gram-negative rods (81.5%), followed by Gram-positive rods (14.8%), and one isolate (3.7%) appeared as spiral-shaped Gram-negative rods. The morphological characteristics of the isolates are presented in Supporting File S2.

The API 20E and Biolog Gen III tests revealed varied biochemical reactions and carbohydrate utilization profiles among the 12 representative Gram-negative isolates (Supporting Files S3a and S3b). Using the API 20E identification system, the isolates were classified into the genera *Enterobacter*, *Klebsiella*, *Pseudomonas*, *Buttiauxella*, and *Pantoea*. In contrast, the Biolog database identified only *Pseudomonas* and *Pantoea* isolates.

### 3.2. Molecular Identification

Pairwise alignment of the 16S rRNA sequences of the isolates resulted in DNA fragments up to 1431 bp. BLASTn results showed that the isolates have high similarities with two or more closely related genera, making this method challenging for obtaining taxonomic assignments. For each isolate, the first three hits with the highest percentage identities (> 96%) were recorded (Supporting File S4).

**Figure 1:**
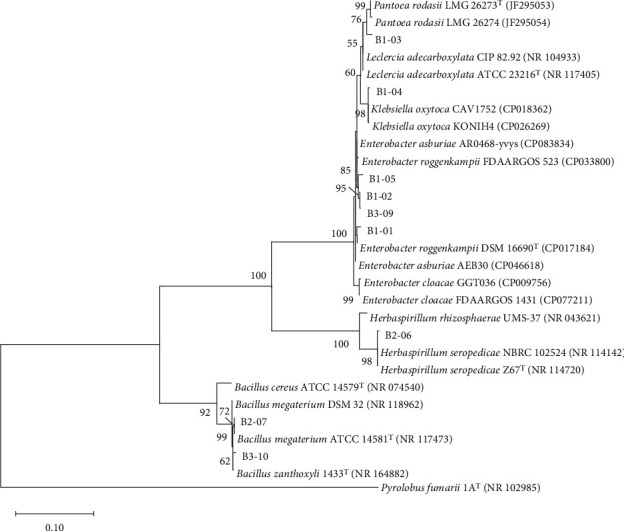
Phylogenetic tree of the nine PGPB strains isolated from bamboo and related strains in GenBank based on 1147 nucleotides of the 16S rRNA gene. The GenBank accession number of each sequence is shown in parentheses. The evolutionary history was inferred using the maximum likelihood method and TN93+G model of DNA substitution. Values on the nodes represent bootstrap percentages out of 1000 bootstrap samples; values < 50% are not shown. The archaea *Pyrolobus fumarii* was used as the outgroup. Scale bar represents substitutions per 100 nucleotides.

**Figure 2:**
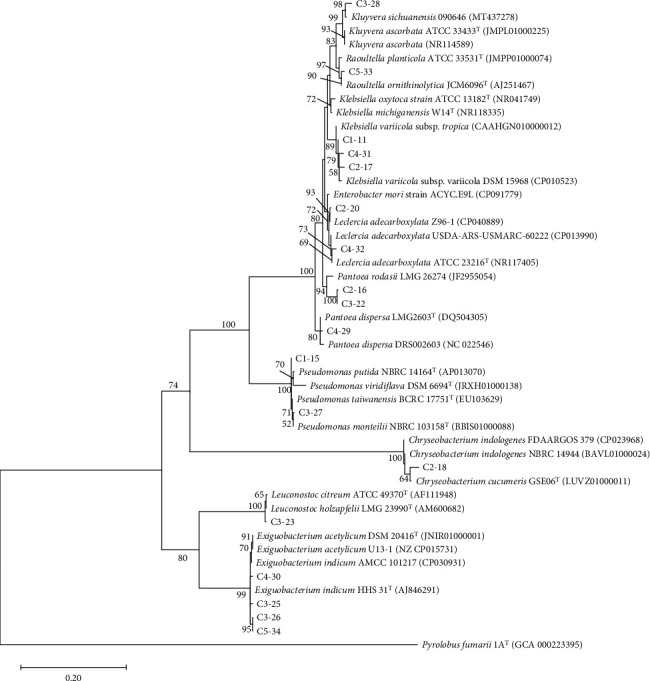
Phylogenetic tree of the 18 corn root–derived PGPB and related strains in GenBank based on 1269 nucleotides of the 16S rRNA gene. The GenBank accession number of each sequence is shown in parentheses. The evolutionary history was inferred using the maximum likelihood method and T92+G model of DNA substitution. Values on the nodes represent bootstrap percentages out of 1000 bootstrap samples; values less than (<) 50% are not shown. The archaea *Pyrolobus fumarii* was used as the outgroup. Scale bar represents substitutions per 100 nucleotides.

Based on the constructed phylogenetic tree of the 16S rRNA gene, bamboo root–derived isolates belonged to five genera under phyla Pseudomonadota (Proteobacteria) and Bacillota (Firmicutes) (Figure 1). On the other hand, isolates from corn were distributed into nine genera under phyla Pseudomonadota, Bacillota, and Bacteroidota (Bacteroidetes) (Figure 2).

Taxonomic determination at the species or genus level was not achieved for some isolates due to the high similarity of 16S rRNA sequences and low bootstrap support in the phylogenetic tree. Hence, three corn root–derived isolates with inconclusive taxonomic assignment, namely, B1-01, B1-02, and B3-09, were selected for WGS. Genomic analysis identified B1-01 as *Enterobacter roggenkampii*, while isolates B1-02 and B3-09 were designated as *Leclercia adecarboxylata*, sharing an average nucleotide identity of 98.5% with each of the reference genomes (Table 1).

The results of the molecular identification methods classified the 27 isolates into 12 genera. The most abundant genus was *L. adecarboxylata* with five isolates. The second most represented genera were *Pantoea*, *Klebsiella*, and *Exiguobacterium*, each with four isolates. *Bacillus* and *Pseudomonas* had two representative isolates each. The genera *Enterobacter*, *Herbaspirillum*, *Chryseobacterium*, *Leuconostoc*, *Kluyvera*, and *Raoultella* were each represented by one isolate (Table 2).

### 3.3. Detection of PGP Traits

Bacterial isolates derived from bamboo and corn roots were screened for PGP capabilities including the presence of *nifH* gene, phosphate solubilization, IAA synthesis, and siderophore production. The results of the screening experiments are presented in Table 2.

#### 3.3.1. Putative N-Fixation Capabilities

All 27 isolates exhibited growth in the different modified N-free broth media and Burk's N-free agar after successive inoculations, suggesting their potential to fix N. To further assess their N-fixation ability, a *nifH* gene–targeting PCR assay was performed on all isolates. The 383-bp fragment of the *nifH* gene was detected in isolates B1-04, C1-11, C2-17, and C4-31, all belonging to the genus *Klebsiella*, as well as in *E. roggenkampii* B1-01, *L. adecarboxylata* B1-02, and *Pseudomonas* sp. C1-15. BLASTX analysis revealed that the deduced sequences from B1-01 and C2-17 shared 90.35% and 93.55% identity, respectively, with a dinitrogenase reductase protein from *Klebsiella pneumoniae*.

#### 3.3.2. Phosphate Solubilization

**Figure 3:**
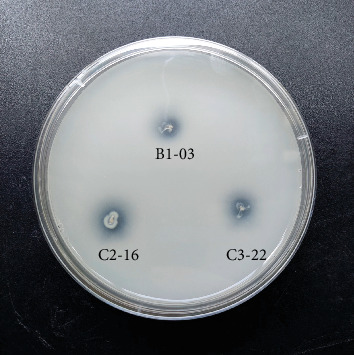
Solubilization of tricalcium phosphate by three *Pantoea rodasii* strains (isolates B1-03, C2-16, and C3-22) after 5 days of incubation on PVK agar.

Eleven isolates showed positive phosphate solubilization activity, indicated by the generation of clearing zones around PVK agar (Figure 3 and Table 2). Among the eleven isolates, four exhibited high solubilization potential, while the remaining ones showed low potential. The most efficient phosphate solubilizers were *Pantoea rodasii* C2-16 (PSI = 8.2), C3-22 (PSI = 5.3), and B1-03 (PSI = 4.4).

#### 3.3.3. IAA Production

Twenty-five strains produced IAA after 72 h of incubation (Table 2). After extending the incubation period up to 144 h, all strains demonstrated the capability to produce IAA at higher concentrations ranging from 4.11 to 221.89 *μ*g/mL. *Klebsiella oxytoca* B1-04 yielded the highest IAA concentration, measuring 221.89 *μ*g/mL, followed by *Chryseobacterium* sp. C2-18 (134.11 *μ*g/mL) and *P. rodasii* C3-22 (108.56 *μ*g/mL).

#### 3.3.4. Siderophore Production

Twenty-four isolates produced siderophores (> 10%) after 72 h of incubation (Table 2). After 144 h, the rate of siderophore production increased for most strains, ranging from 19.42% to 88.59%. However, the number of siderophore-positive isolates was reduced to 22. The highest siderophore producers were *Raoultella ornithinolytica* C5-33 (88.59% siderophore production units), *L. adecarboxylata* B1-02 (87.62%), and *L. adecarboxylata* B3-09 (87.38%). Meanwhile, *P. rodasii* strains, namely, C2-16 and C3-22, and *Leuconostoc* sp. C3-23 did not produce siderophores.

#### 3.3.5. Multifunctional Potential of PGPB Isolates

**Figure 4:**
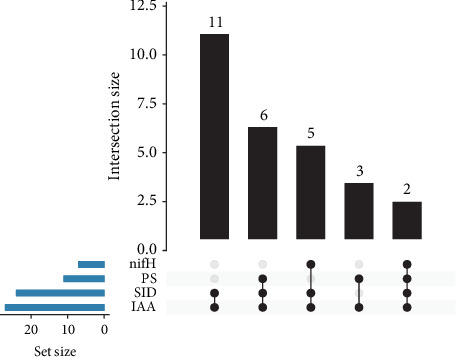
UpSet plot of PGP traits detected by biochemical assays and by PCR. The horizontal bar chart on the left indicates the total number of isolates that exhibit each PGP trait. The vertical bar chart indicates the intersection size between sets of isolates with one or more PGP traits. Connected black dots indicate which PGP trait is considered for each intersection.

As depicted in Table 2 and Figure 4, all isolates demonstrated positive results for at least two PGP traits. Eleven (11) isolates were capable of producing both IAA and siderophores, while six strains demonstrated three PGP traits, including phosphate solubilization, IAA production, and siderophore production. Moreover, five isolates tested positive for the *nifH* gene, IAA production, and siderophore production while three isolates were found to solubilize phosphate and produce IAA. Notably, two isolates, identified as *E. roggenkampii* B1-01 and *K. oxytoca* B1-04, exhibited all four tested PGP traits.

**Figure 5:**
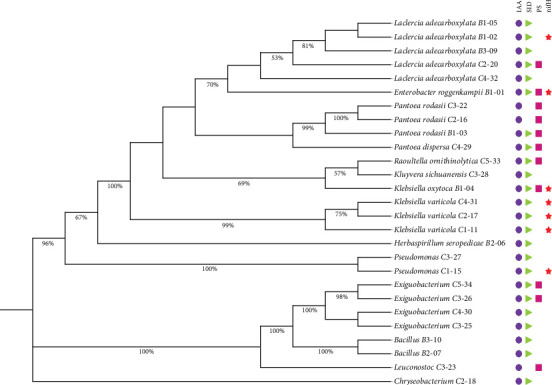
Diversity of PGP traits and phylogenetic relationships among 27 PGPB isolates based on 1202 nucleotides of the 16S rDNA sequences. The evolutionary history was inferred using the maximum likelihood method and TN93+G model of DNA substitution. Values on the nodes represent bootstrap percentages out of 1000 bootstrap samples; values less than (<) 50% are not shown. Production of indole-3-acetic acid (purple circle), siderophore production (green triangle), phosphate solubilization (pink square), and detection of the *nifH* gene (red star).

Additionally, a phylogenetic tree of the isolates alongside their PGP attributes was created (Figure 5). Genera with the most number of representatives, particularly, *Leclercia*, *Pantoea*, and *Exiguobacterium*, showed diversity in PGP traits. *Exiguobacterium* isolates were categorized as phosphate solubilizers and nonphosphate solubilizers. Interestingly, isolate *K. oxytoca* B1-04, which was positive for all growth-promoting tests, is phylogenetically separated from the *Klebsiella variicola* group which were nonphosphate solubilizers suggesting that some traits may be specific to bacterial species.

## 4. Discussion

### 4.1. Taxonomic Characterization of the Isolated Strains

Majority of the root-associated bacteria (70.4%) isolated in this study were classified under classes Beta- and Gammaproteobacteria and phylum Pseudomonadota (Proteobacteria). The prevalence of Proteobacteria in the rhizosphere of bamboo and corn has been documented in previous studies [43–45]. Members of the Enterobacteriaceae family such as *Leclercia*, *Pantoea*, and *Klebsiella* were the most represented among the PGPB isolates. The prevalence of *Leclercia*, such as *L. adecarboxylata*, in rhizospheres of plants, including corn, has been reported [46]. Kang et al. [47] isolated IAA and ACC deaminase–producing *L. adecarboxylata* MO1 from tomato rhizosphere which improved the growth of tomato under salt stress. *Pantoea* was previously isolated from crops belonging to the grass family, such as corn, sugarcane, and wheat with documented high nutrient mineralization and biocontrol activities [48–51]. *Klebsiella* is also a frequently isolated bacteria from the rhizosphere of graminaceous crops [52]. Certain species of *Klebsiella* (*K. oxytoca*, *K. pneumoniae*, and *Klebsiella planticola*) were identified as associative N-fixing bacteria, and some strains produce copious amounts of various PGP [53, 54]. Members of the less represented genera within the phylum Pseudomonadota, such as *Enterobacter*, *Pseudomonas*, and *Herbaspirillum*, have been commonly reported as endophytic and rhizospheric PGPB [17, 53]. In contrast, *Kluyvera* and *Raoultella* have been less frequently reported as endophytic PGPB [33, 55].

Phylum Bacillota is the second most well-represented phylum among the PGPB isolates in this study (26%), represented by the genera *Exiguobacterium*, *Bacillus*, and *Leuconostoc*. *Exiguobacterium* spp. have been known to thrive in harsh environments but their role as a plant growth promoter is underexplored [56]. Meanwhile, *Bacillus* is a common PGPB frequently isolated from the rhizosphere of both bamboo and corn [14–16]. One corn root–derived isolate was identified as *Chryseobacterium*, which belongs to phylum Bacteroidota. Species of *Chryseobacterium* were isolated from various environments including the rhizosphere of several plants, such as corn [57], and have been shown to have activities in plant growth promotion or biocontrol of plant pathogens [58–60].

### 4.2. PGP Activities of the Isolates

Nitrogen-fixing bacteria (NFB) play a critical role in plant nutrition by converting atmospheric nitrogen (N_2_) to ammonia (NH_3_), a form that plants can readily utilize. These bacteria possess nitrogenase, the enzyme responsible for N fixation. The *nifH* gene is one of the oldest existing and functioning genes in the history of gene evolution and showed consistency with 16S rRNA phylogeny, offering valuable insights for studies on diazotrophic biodiversity [61]. While the growth on N-free media suggests the diazotrophic potential of the isolates, detection of the *nifH* gene further substantiates the N-fixing capabilities of the candidate PGPB. A small percentage of *nifH*-positive strains (25.9%) were identified in this study such as *Klebsiella*, *Pseudomonas*, *Leclercia*, and *Enterobacter*. Earlier studies documented strains within these above-mentioned genera that display the capacity for N fixation [53, 62, 63]. According to Kuklinsky-Sobral et al. [64] and Zehr et al. [65], other symbiotic N-fixing bacteria may have variable *nifH* gene sequences which could not be amplified by the current PCR protocol.

P is the second most important nutrient for plant growth and development after N, since it is a building block for the synthesis of nucleic acids for all living organisms. Phosphate-solubilizing bacteria (PSB) release organic acids that solubilize insoluble forms of phosphate in the soil making it utilizable to plants. Interestingly, the three most efficient PSB in this study (B1-03, C2-16, and C3-22) were all identified as *P. rodasii*. Several strains of *Pantoea* sp. isolated from different environments were able to demonstrate good phosphate solubilization [66, 67] and increased yield of corn plants [68]. Chen and Liu [69] isolated PSB strains from a heavy-metal contaminated reclamation soil, and they identified *Pantoea* sp. S32 with the highest P solubilization efficiency. Phylogenetic analysis of S32 showed its close association with *P. rodasii*. These findings suggest the potential of *P. rodasii* as a candidate for highly efficient PSB.

IAA, also known as auxin, is a phytohormone known to promote root elongation and plant growth [70] even when under biotic and abiotic stress [71, 72]. Root elongation enhances plant mineral uptake and exudation which would also increase bacterial inoculation by expanding the rhizosphere [73]. In this study, all isolates produced IAA in varying amounts. Moreover, the levels of IAA considerably increased after a longer period of incubation. The most efficient IAA producers include *K. oxytoca* B1-04 (221.89 *μ*g/mL), *Chryseobacterium* sp. C2-18 (134.11 *μ*g/mL), and *P. rodasii* C3-22 (108.56 *μ*g/mL). The levels of IAA produced by the first two strains were considerably higher than those documented in previous studies [59, 74]. Meanwhile, IAA production of *P. rodasii* was recorded to reach up to 229 *μ*g/mL in optimized conditions [75]. The variation in IAA synthesis of PGPB isolated from bamboo and corn rhizosphere was also observed in previous studies [15, 16]. It has been reported that IAA production in vitro is dependent on bacterial species and strains, substrate availability, and various pathways for bacterial biosynthesis of IAA [76].

Microorganisms and plants produce iron-chelating compounds called siderophores which are used to scavenge Fe^3+^ ions from insoluble minerals in the soil environment; thus, under iron-deficient conditions, iron availability is enhanced [77]. Fe^3+^ is then transported to the cell and converted into a biologically suitable ferrous [Fe^2+^] form [78]. Aside from chelating ferric ions, siderophores are reported to inhibit colonization of the roots by plant pathogens [79, 80] which makes them suitable biocontrol agents [81]. Siderophores can be also utilized for bioremediation of heavy metal–contaminated environments [78]. Results showed that siderophore production was common to all genera identified in this study except for *Leuconostoc* and some species of *Pantoea*. Various studies previously reported siderophore production *in Enterobacter*, *Klebsiella*, *Pantoea*, *Pseudomonas*, *Exiguobacterium*, and *Bacillus* strains [82–85]. The ability of the bacterial genera or species to produce siderophores at highly variable rates can be attributed to different intrinsic capacities of strains and environmental factors such as pH, temperature, and carbon sources [86].

The rhizosphere, defined as the plant root–soil interface, has the most dynamic interactions due to its high microbial activity and diversity [87]. Studies have harnessed the beneficial plant–microbe interactions to develop inoculants containing single or consortia of PGPB to improve plant health. For instance, Mowafy et al. [85] demonstrated that interactions between endophytic bacteria *Bacillus*, *Enterobacter*, and *Klebsiella* resulted in better nutrient uptake of corn seedlings due to the increased number of lateral roots and inhibition of ethylene, which resulted in increased growth and yield compared to untreated plants in the field. Similarly, Zhao et al. [88] found that *Burkholderia pyrrocinia* BD24-2, isolated from moso bamboo shoots, significantly promoted moso bamboo seedling growth in pot experiments by promoting root hair growth. These findings suggest that bamboo and corn roots harbor numerous beneficial bacteria that act in synergy to contribute to their health and productivity. In this preliminary study, several promising strains of PGPB were isolated, which could be further investigated to assess their direct effects on plant growth.

### 4.3. Biosafety of PGPB

In this study, some of the isolated PGPB belonging to the genera *Leclercia*, *Klebsiella*, *Enterobacter*, and *Herbaspirillum* have previously been identified as opportunistic pathogens [89–91]. Given the potential risks associated with the use of opportunistic PGPB in the field, researchers suggested implementing a safety evaluation of the strains through several steps. These include the determination of the genotypic and phenotypic traits related to virulence factors and antimicrobial resistance, as well as in vitro and in vivo testing of pathogenic potential in plants and animals [92, 93]. For example, genomic analysis of endophytic PGPB *Klebsiella michiganensis* Kd70, a species belonging to the *K. oxytoca* species complex, revealed the absence of pathogen island–like regions and fewer mammalian virulence factors compared to clinical isolates of the same species [94]. In vivo experiments also showed that Kd70 was unable to invade the mouse urinary tract, signifying its potential to be developed as a biofertilizer without major health risks. In *K. pneumoniae*, the presence of N-fixation genes is thought to support adaptation to plant hosts, whereas the absence of this capability in clinical isolates likely contributed to their evolution into human pathogens [95]. Taken together, these studies highlight the importance of subjecting each candidate strain to a standard battery of laboratory tests to ensure its biosafety.

## 5. Conclusions

This study documented the presence of diverse PGPB in the rhizosphere of agriculturally important crops such as bamboo and corn. Based on 16S rRNA and WGS analyses, the 27 isolates were classified into 12 genera dominated by *Leclercia*, *Pantoea*, *Klebsiella*, and *Exiguobacterium*. The strains were also found to exhibit two or more PGP traits based on the assays detecting the *nifH* gene, phosphate solubilization, IAA production, and siderophore production. The most notable strains are *E. roggenkampii* strain B1-01 and *K. oxytoca* B1-04 displaying all the tested PGP traits. Strains from different genera were also identified as capable of producing substantial quantities of either IAA or siderophores or exhibiting high phosphate solubilization potential. However, thorough biosafety assessments of promising strains must be conducted before they can be selected for future studies as microbial inoculants.

## Figures and Tables

**Table 1:** Genome features and classification of selected PGPB strains.

**Genome characteristics**	**B1-01**	**B1-02**	**B3-09**
Genome size (bp)	4,602,963	4,904,472	4,902,885
GC content (%)	56	57	57
FastANI (%)	98.5	98.5	98.5
FastANI reference	GCF_001729805.1	GCF_006171285.1	GCF_006171285.1
Classification	*Enterobacter roggenkampii*	*Leclercia adecarboxylata*	*Leclercia adecarboxylata*

Abbreviation: ANI, average nucleotide identity.

**Table 2:** Plant growth–promoting properties and molecular identification of the isolates based on 16S rRNA sequence–based phylogenetic analysis or whole-genome sequencing (WGS).

**Isolate code**	***nifH*** ^**a**^	**PSI**	**IAA (*μ*g/mL)**		**SID (%)**		**Taxonomic identification**
			**72** h	**144** h	**72 h**	**144 h**	
Bamboo root–derived isolates							
B1-01	+^b^	1.2	35.11	86.33	75.48	79.85	*Enterobacter roggenkampii* ^d^
B1-02	+	−^c^	5.11	16.33	31.92	87.62	*Leclercia adecarboxylata* ^d^
B1-03	−	4.4	56.68	27.44	15.01	7.04	*Pantoea rodasii*
B1-04	+	1.3	37.74	221.89	59.62	86.89	*Klebsiella oxytoca*
B1-05	−	−	6.16	5.22	36.36	83.01	*Leclercia adecarboxylata*
B2-06	−	−	35.11	68.56	44.82	40.05	*Herbaspirillum seropedicae*
B2-07	−	−	8.79	17.44	39.53	49.27	*Bacillus* sp.
B3-09	−	−	6.68	27.44	35.31	87.38	*Leclercia adecarboxylata* ^d^
B3-10	−	−	13	60.78	35.73	44.9	*Bacillus* sp.
Corn root–derived isolates							
C1-11	+	−	6.68	25.22	59.2	87.14	*Klebsiella variicola*
C1-15	+	−	29.84	35.22	65.54	74.76	*Pseudomonas* sp.
C2-16	−	8.2	17.74	61.89	4.44	7.28	*Pantoea rodasii*
C2-17	+	−	6.16	18.56	69.98	83.74	*Klebsiella variicola*
C2-18	−	−	83	134.11	40.38	46.84	*Chryseobacterium* sp.
C2-20	−	1.6	−5.42	16.33	22.62	82.77	*Leclercia adecarboxylata*
C3-22	−	5.3	7.74	108.56	9.51	7.77	*Pantoea rodasii*
C3-23	−	1.5	6.68	29.67	10.36	4.85	*Leuconostoc* sp.
C3-25	−	−	3.79	25.22	−4.02	32.52	*Exiguobacterium* sp.
C3-26	−	4.2	59.32	39.67	13.95	19.42	*Exiguobacterium* sp.
C3-27	−	−	76.16	51.89	34.25	60.68	*Pseudomonas* sp.
C3-28	−	−	4.05	30.78	76.32	83.01	*Kluyvera sichuanensis*
C4-29	−	1.4	13.53	60.78	21.35	−7.04	*Pantoea dispersa*
C4-30	−	−	7.74	24.11	10.57	29.37	*Exiguobacterium* sp.
C4-31	+	−	8.79	28.56	32.35	70.39	*Klebsiella variicola*
C4-32	−	−	4.05	13	42.28	33.98	*Leclercia* sp.
C5-33	−	1.5	0.89	31.89	79.7	88.59	*Raoultella ornithinolytica*
C5-34	−	1.3	−6.47	4.11	17.12	22.57	*Exiguobacterium* sp.

Abbreviations: IAA = indole-3-acetic acid production; PSI = phosphate solubilization index; SID = siderophore production.

^a^PCR detection of the *nifH* gene.

^b^+ indicates positive result.

^c^− indicates negative result.

^d^WGS-based identification.

## Data Availability

The data used to support the findings of this study are included within the supporting information files.

## Supporting Information

Supporting Information 1 Supporting File S1: Nitrogen-free basal media based on Dr. Peter Jurtshuk.

Supporting Information 2 Supporting File S2: Colony and cellular characteristics of 48-h-old PGPB isolates on Burk's medium agar.

Supporting Information 3 Supporting File S3a: Biochemical and carbohydrate utilization profile of selected Gram-negative isolates based on API 20E test kit.

Supporting Information 4 Supporting File S3b: Biochemical and carbohydrate utilization profile of selected Gram-negative isolates based on Biolog Gen III identification system.

Supporting Information 5 Supporting File S4: Molecular identification of the 27 bacteria isolated from bamboo and corn roots through 16S rRNA sequencing.
