# Supplementary material for: Multiple Plant Growth–Promoting Activities Exhibited by Root-Associated Bacteria Isolated From Bamboo and Corn
Source: Int J Microbiol. 2025 Mar 11;2025:6374935. doi: 10.1155/ijm/6374935 (PMC11987075; doi:10.1155/ijm/6374935)
Supplement: Supporting Information 1 — Supporting File S1: Nitrogen-free basal media based on Dr. Peter Jurtshuk. [file 6374935.f1.pdf]

Supplementary File S1. Nitrogen-free basal media based on Dr. Peter Jurtshuk (1988)

1. **NF modified 5 Basal Medium (NF5)**

Components	Ingredients (g per 1 L of distilled water)
di-Potassium hydrogen phosphate	0.30
Potassium dihydrogen orthophosphate monobasic	0.70
Magnesium sulphate	0.20
Ferrous sulphate	0.05
Sodium molybdate	0.01
Sodium bicarbonate	0.05
Strontium chloride	0.02
DL-Malic Acid	5.00
Yeast extract	0.05
Glucose	18.00
Sucrose	2.00
L-Tryptophan	0.05
Agar	15.00

2. NF modified 6 Basal Medium (NF6) with 1% glucose

Components	Ingredients (g per 1 L of distilled water)
di-Potassium hydrogen phosphate	1.00
Magnesium sulphate	0.20
Calcium chloride	0.20
Ferrous sulphate	0.05
Sodium molybdate	0.01
Sodium bicarbonate	0.05
Strontium chloride	0.02
DL-Malic Acid	5.00
Yeast extract	0.05
Glucose	10.00
L-Tryptophan	0.05
Agar	15.00

3. NF modified 6 Basal Medium (NF6) with 2% glucose

Components	Ingredients (g per 1 L of distilled water)
di-Potassium hydrogen phosphate	1.00
Magnesium sulphate	0.20
Calcium chloride	0.20
Ferrous sulphate	0.05
Sodium molybdate	0.01
Sodium bicarbonate	0.05
Strontium chloride	0.02
DL-Malic Acid	5.00
Yeast extract	0.05
Glucose	20.00
L-Tryptophan	0.05
Agar	15.00

4. NF (mod 8) Basal Medium (NF8)

Components	Ingredients (g per 1 L of distilled water)
di-Potassium hydrogen phosphate	1.00
Magnesium sulphate	0.20
Sodium chloride	0.20
Calcium chloride	0.10
Ferrous sulphate	0.05
Sodium molybdate dihydrate	0.01
Sodium bicarbonate	0.05
Strontium chloride	0.02
DL-Malic Acid	5.00
Yeast extract	0.05
Glucose	18.00
Mannitol	2.00
L-Tryptophan	0.05
Agar	15.00
