# Supplementary material for: Multiple Plant Growth–Promoting Activities Exhibited by Root-Associated Bacteria Isolated From Bamboo and Corn
Source: Int J Microbiol. 2025 Mar 11;2025:6374935. doi: 10.1155/ijm/6374935 (PMC11987075; doi:10.1155/ijm/6374935)
Supplement: Supporting Information 2 — Supporting File S2: Colony and cellular characteristics of 48-h-old PGPB isolates on Burk's medium agar. [file 6374935.f2.pdf]

Supplementary File S2. Colony and cellular characteristics of 48hr-old PGPB isolates on Burk's medium agar

ISOLATE CODE	FORM	SIZE	MARGIN	SURFACE	OPACITY	PIGMENTATION	GRAM REACTION
Bamboo isolates							
B1-01	round	0.5-1.0	entire	glistening	translucent	white	G (-) rods
B1-02	round	2.5-3.0	entire	glistening	opaque	light yellow	G (-) rods
B1-03	round	1.0-2.5	entire	smooth	translucent	light yellow	G (-) rods
B1-04	round	1.4-2.3	entire	glistening	translucent	white	G (-) rods
B1-05	round	1.9-2.7	entire	glistening	opaque	white	G (-) rods
B2-06	round	1.0	entire	glistening	translucent	white	G (-) helical rods
B2-07	round	3.0-4.0	entire	glistening	opaque	light yellow	G (+) rods
B3-09	round	0.5	entire	smooth	opaque	white	G (-) rods
B3-10	round	1.7-3.27	entire	glistening	opaque	white	G (+) rods
Corn isolates							
C1-11	round	3.0	entire	glistening	translucent	white	G (-) rods
C1-15	round	0.5	entire	glistening	opaque	white	G (-) rods
C2-16	round	0.5	entire	glistening	translucent	white	G (-) rods
C2-17	round	3.7	entire	glistening	translucent	white	G (-) rods with capsule
C2-18	round	0.8-1.4	entire	glistening	opaque	dark yellow	G (-) rods
C2-20	round	0.6-0.8	entire	glistening	opaque	light yellow	G (-) rods
C3-22	round	0.6-0.8	entire	glistening	opaque	white	G (-) rods
C3-23	round	7.3-7.8	entire	glistening	transparent	colorless	G (+) rods
C3-25	round	0.5-0.8	entire	smooth	opaque	yellow	G (+) rods
C3-26	round	1.5-3.0	entire	glistening	transparent	colorless	G (+) rods
C3-27	round	1.0	entire	glistening	translucent	white	G (-) rods
C3-28	round	2.3-2.4	entire	glistening	opaque	white	G (-) rods
C4-29	round	spreading	entire	mucoid	translucent	white	G (-) rods
C4-30	round	0.8	entire	glistening	opaque	light orange	G (+) rods
C4-31	round	3.6-4.0	entire	glistening	translucent	white	G (-) rods with capsule
C4-32	round	0.7-1.0	entire	glistening	translucent	light yellow	G (-) rods
C5-33	round	3.8-4.7	entire	glistening	opaque	white	G (-) rods
C5-34	round	1.7-2.1	entire	glistening	opaque	light orange	G (+) rods
