# Supplementary material for: Multiple Plant Growth–Promoting Activities Exhibited by Root-Associated Bacteria Isolated From Bamboo and Corn
Source: Int J Microbiol. 2025 Mar 11;2025:6374935. doi: 10.1155/ijm/6374935 (PMC11987075; doi:10.1155/ijm/6374935)
Supplement: Supporting Information 3 — Supporting File S3a: Biochemical and carbohydrate utilization profile of selected Gram-negative isolates based on API 20E test kit. [file 6374935.f3.pdf]

Supplementary file S3a. Biochemical and carbohydrate utilization profile of selected Gram-negative isolates based on API 20E test kit.

	ISOLATES TESTED											
**Tests**	**B1-01**	**B1-02**	**B1-03**	**B1-04**	**C1-11**	**C1-15**	**C2-16**	**C2-17**	**C2-20**	**C4-29**	**C4-31**	**C5-33**
ß-galactosidase	+	+	+	+	+	-	+	+	+	+	+	+
Arginine dihydrolase	+	-	-	-	-	+	-	+	-	-	-	-
Lysine decarboxylase	-	-	-	+	+	-	-	+	-	-	+	+
Ornithine decarboxylase	+	+	-	-	-	-	-	-	+	-	-	-
Citrate utilization	+	+	+	+	+	+	+	+	+	+	+	+
H_2S production	-	-	-	-	-	-	-	-	-	-	-	-
Urease	-	-	-	-	+	-	-	+	-	-	-	-
Tryptophane deaminase	-	-	+	-	-	+	+	-	+	-	-	+
Indole production	-	-	-	-	-	-	-	-	-	-	-	-
Acetoin production (Voges Proskauer)	+	-	+	+	+	-	-	-	-	+	+	+
Gelatinase	-	-	-	-	-	-	-	-	-	-	-	-
Fermentation / oxidation glucose	+	+	+	+	+	+	+	-	-	+	-	-
mannitol	+	+	+	+	+	-	+	+	+	+	+	+
inositol	-	-	-	+	+	-	-	+	-	-	+	-
sorbitol	+	+	-	+	+	-	-	+	+	-	+	+
rhamnose	-	+	+	+	+	-	+	+	+	+	+	+
saccharose	+	-	-	+	+	-	-	+	-	+	+	+
melibiose	+	+	-	+	-	-	-	+	+	+	+	+
amygdalin	+	+	+	+	+	-	+	+	+	-	+	+
arabinose	+	+	+	+	+	-	+	+	+	+	+	+
Cytochrome-oxidase	-	-	-	-	-	+	-	-	-	-	-	-
