# Supplementary material for: Multiple Plant Growth–Promoting Activities Exhibited by Root-Associated Bacteria Isolated From Bamboo and Corn
Source: Int J Microbiol. 2025 Mar 11;2025:6374935. doi: 10.1155/ijm/6374935 (PMC11987075; doi:10.1155/ijm/6374935)
Supplement: Supporting Information 4 — Supporting File S3b: Biochemical and carbohydrate utilization profile of selected Gram-negative isolates based on Biolog Gen III identification system. [file 6374935.f4.pdf]

Supplementary File S3b: Biochemical and carbohydrate utilization profile of selected Gram-negative isolates based on Biolog GenIII identification system. The plus (+), capital letter (W), and minus (–) show the positive, weak, and negative reactions of strains on a substrate, respectively.

	ISOLATES TESTED											
**Biochemical tests**	**B1-01**	**B1-02**	**B1-03**	**B1-04**	**C1-11**	**C1-15**	**C2-16**	**C2-17**	**C2-20**	**C4-29**	**C4-31**	**C5-33**
**Substrate utilization**												
Dextrin	+	-	-	W	+	-	-	+	-	-	+	W
D-Maltose	-	+	-	+	-	-	-	+	+	-	W	-
D-Trehalose	+	+	W	+	-	-	W	+	+	W	+	-
D-Cellobiose	+	W	W	+	-	-	-	+	+	-	+	-
Gentiobiose	+	+	+	+	-	W	+	+	+	W	+	-
Sucrose	+	-	-	+	-	-	-	+	-	W	+	-
D-Turanose	-	W	W	+	-	W	-	+	W	+	-	W
Stachyose	-	-	-	+	+	-	-	+	-	-	-	-
D-Raffinose	+	+	+	+	-	-	-	+	+	-	+	-
D-Lactose	+	-	-	-	-	-	-	+	W	-	-	W
D-Melibiose	+	W	+	+	-	-	W	+	+	W	W	-
β-Methyl-D-Glucoside	+	+	W	+	-	-	W	+	W	-	+	-
D-Salicin	+	-	W	+	+	-	W	+	+	+	+	-
N-Acetyl-D-Glucosamine	+	-	+	+	+	-	W	+	+	+	+	+
N-Acetyl-β-DMannosamine	+	-	+	+	+	-	W	+	+	+	+	+
N-Acetyl-DGalactosamine	-	+	-	-	+	-	-	-	-	-	-	W
N-Acetyl Neuraminic Acid	-	+	-	-	+	-	-	-	+	-	-	+
D-Glucose	+	W	+	+	-	W	+	+	+	W	+	-
D-Mannose	+	W	+	+	-	+	W	+	+	+	+	-
D-Fructose	+	+	+	+	-	-	W	+	+	W	+	-
D-Galactose	+	+	+	+	+	W	+	+	+	+	+	-
3-Methyl Glucose	+	-	W	-	+	W	+	-	-	W	-	-
D-Fucose	-	-	+	W	+	W	+	+	-	+	+	-
L-Fucose	-	-	W	+	+	-	W	+	-	-	+	-
L-Rhamnose	+	+	+	-	-	-	+	+	+	+	-	-
Inosine	-	+	W	W	-	W	W	+	+	+	-	-
D-Sorbitol	+	+	W	+	-	-	W	+	+	-	+	-
D-Mannitol	+	W	W	+	-	-	W	+	+	-	+	-
D-Arabitol	-	-	+	+	-	-	+	+	-	W	-	-
Myo-inositol	-	-	+	-	+	-	+	+	-	+	-	-

Glycerol	+	+	+	+	-	W	+	-	+	+	-	-
D-Glucose- 6-PO4	-	+	+	+	+	-	W	+	+	+	+	+
D-Fructose- 6-PO4	+	+	+	+	+	-	W	+	+	-	+	+
D-Aspartic Acid	-	-	-	-	+	-	-	-	-	-	-	-
D-Serine	-	+	-	+	+	-	-	+	+	-	+	+
Gelatin	-	-	-	-	+	-	-	-	-	-	-	-
Glycyl-L-Proline	-	+	+	-	+	-	+	+	+	+	-	-
L-Alanine	-	+	+	+	+	+	+	+	+	+	-	+
L-Arginine	-	W	+	W	+	-	W	+	W	-	+	+
L-Aspartic Acid	-	+	+	+	+	+	+	+	+	+	-	+
L-Glutamic Acid	-	+	+	+	+	+	+	+	+	+	+	+
L-Histidine	-	+	+	+	+	+	+	+	+	+	W	+
L-Pyroglutamic Acid	-	-	-	-	-	+	-	-	-	-	-	-
L-Serine	-	+	+	+	+	W	+	+	+	+	-	-
Pectin	-	-	-	+	+	-	-	+	-	+	+	W
D-Galacturonic Acid	W	+	+	+	+	+	+	+	+	+	+	+
L-Galactonic Acid Lactone	-	+	+	+	+	+	+	+	+	+	+	+
D-Gluconic Acid	+	+	+	+	+	+	+	+	+	+	+	+
D-Glucuronic Acid	-	+	+	+	+	+	+	+	+	+	+	+
Glucuronamide	-	+	+	+	+	+	+	+	+	+	+	-
Mucic Acid	-	+	+	+	+	+	+	+	+	+	+	+
Quinic Acid	-	-	+	+	+	+	+	+	-	+	+	+
D-Saccharic Acid	-	+	+	+	+	+	+	+	+	+	+	+
p-Hydroxy- Phenylacetic Acid	-	+	-	-	-	-	-	+	+	-	-	-
Methyl Pyruvate	-	+	W	+	-	-	W	+	+	-	-	-
D-Lactic Acid Methyl Ester	-	W	-	-	+	-	-	-	W	-	-	W
L-Lactic Acid	-	+	+	+	+	+	+	+	+	+	+	W
Citric Acid	-	+	+	+	+	+	+	+	+	+	+	+
α-Keto-Glutaric Acid	-	-	-	-	-	-	-	-	-	-	-	-
D-Malic Acid	-	-	+	+	+	-	+	+	-	W	-	W
L-Malic Acid	+	+	+	+	+	+	+	+	+	+	+	+
Bromo-Succinic Acid	+	+	+	-	+	+	+	+	+	+	-	W
Tween 40	-	-	-	-	-	W	-	-	-	W	-	-
γ-Amino-Butyric Acid	-	-	+	-	+	-	+	-	-	+	W	+
α-HydroxyButyric Acid	-	-	-	-	-	-	-	-	-	-	-	-

β-Hydroxy-D,LButyric Acid	-	-	W	-	+	-	W	+	-	-	-	W
α-Keto-Butyric Acid	+	-	-	-	-	-	-	-	-	-	-	-
Acetoacetic Acid	-	W	-	-	-	-	-	-	-	-	-	-
Propionic Acid	-	-	-	-	-	-	-	-	-	W	-	-
Acetic Acid	-	+	W	W	-	+	+	+	+	+	-	+
Formic Acid	-	W	+	W	-	W	W	-	-	+	-	-
**Chemical Sensitivity**												
pH 6	+	+	+	+	+	+	+	+	+	+	+	+
pH 5	-	W	-	-	W	+	-	-	-	+	-	W
1% NaCl	+	+	+	+	+	+	+	+	+	+	+	+
4% NaCl	-	+	-	+	+	+	-	+	+	+	+	+
8% NaCl	-	-	-	-	-	-	-	-	-	+	-	W
1% Sodium Lactate	W	+	+	W	+	+	+	+	+	+	+	+
Fusidic Acid	-	-	+	-	+	+	-	-	-	W	-	-
D-Serine	-	-	-	-	-	-	-	-	+	-	-	+
Troleandomycin	-	+	+	+	+	+	+	+	+	W	+	+
Rifamycin SV	-	+	+	+	+	+	+	+	+	+	+	+
Minocycline	-	-	-	-	-	+	-	-	-	-	-	-
Lincomycin	-	+	-	+	+	+	-	+	+	+	+	+
Guanidine HCl	-	+	W	+	+	+	+	+	+	+		+
Niaproof 4	-	+	+	+	+	+	+	+	+	+	+	+
Vancomycin	-	+	+	+	+	+	+	+	+	+	+	+
Tetrazolium Violet	-	+	+	+	+	+	+	+	+	+	+	+
Tetrazolium Blue	-	+	+	+	+	+	+	+	+	+	+	+
Nalidixic Acid	-	-	-	-	-	+	-	-	-	-	-	-
Lithium Chloride	+	-	W	+	+	+	-	+	+	W	+	+
Potassium Tellurite	-	-	-	-	-	+	-	+	-	-	+	-
Aztreonam	-	-	-	-	+	+	-	+	+	-	+	+
Sodium Butyrate	-	+	-	+	+	+	-	+	+	+	+	+
Sodium Bromate	-	-	-	-	-	-	-	-	-	-	-	-
