# Supplementary material for: Multiple Plant Growth–Promoting Activities Exhibited by Root-Associated Bacteria Isolated From Bamboo and Corn
Source: Int J Microbiol. 2025 Mar 11;2025:6374935. doi: 10.1155/ijm/6374935 (PMC11987075; doi:10.1155/ijm/6374935)
Supplement: Supporting Information 5 — Supporting File S4: Molecular identification of the 27 bacteria isolated from bamboo and corn roots through 16S rRNA sequencing. [file 6374935.f5.pdf]

Supplementary File S4. Molecular identification of the 27 bacteria isolated from bamboo and corn roots through 16S rRNA sequencing. The first hits in the BLASTn database with >96% identity are presented and were considered for possible identification.

ISOLATE	16S rDNA fragment size (bp)	Possible ID	Closely Related Taxa (BLASTn)	% Identity	Accession No.
B1-01	1306	*Enterobacter*	*Enterobacter roggenkampii*	96.63%	MT256259.1
			Enterobacter mori	96.55%	MT613379.1
			Enterobacter ludwigii	96.55%	MT613360.1
			Enterobacter cloacae	96.55%	MN932286.1
B1-02	1400	*Enterobacter / Leclercia*	*Leclercia* sp.	99.71%	MW205899.1
			Enterobacter sp.	99.64%	KP720604.1
			Enterobacter sichuanensis	99.57%	MG832788.1
B1-03	1402	*Enterobacter / Pantoea*	*Pantoea rodasii*	99.21%	MG516176.1
			Enterobacter sp.	99.00%	KP145012.1
			Pantoea rodasii	99.14%	MG571723.1
B1-04	1298	*Enterobacter / Klebsiella*	*Klebsiella oxytoca*	98.92%	KC462193.1
			Klebsiella sp.	98.92%	LC484748.1
			Enterobacter sp.	98.92%	KT860062.1
B1-05	1247	*Enterobacter / Leclercia*	*Enterobacter sp.* IPC1-8	99.20%	KP720604.1
			Leclercia sp.	99.20%	MW205899.1
			Enterobacter sp.	99.12%	MT321506.1
B2-06	1342	*Herbaspirillum seropedicae*	*Herbaspirillum seropedicae*	99.70%	CP011930.1
			Herbaspirillum seropedicae	99.70%	NR_114142.1
B2-07	1231	*Bacillus megaterium*	*Bacillus megaterium*	99.51%	MK966390.1
			Bacillus sp.	99.51%	GQ406746.1
			Bacillus megaterium	99.51%	MT103023.1
B3-09	1349	*Enterobacter / Leclercia*	*Leclercia* sp.	99.11%	MW205899.1

			Enterobacter sp.	99.04%	KP720604.1
B3-10	1086	*Bacillus*	*Bacillus aryabhattai*	99.54%	KP342174.1
			Priestia megaterium	99.54%	CP085440.1
			Bacillus zanthoxyli	99.54%	ON693697.1
C1-11	1177	*Klebsiella*	*Klebsiella variicola*	99.92%	MN428217.1
			Klebsiella sp.	99.92%	KR063540.1
			Klebsiella pneumoniae	99.83%	MN512286.1
C1-15	1276	*Pseudomonas*	*Pseudomonas* sp.	99.45%	MT386178.1
			Pseudomonas viridiflava	99.45%	MT386130.1
			Pseudomonas putida	99.45%	MF348181.1
			Pseudomonas taiwanensis	99.45%	KM817286.1
C2-16	1313	*Enterobacter*	*Enterobacter* sp.	99.24%	MN540105.1
			Enterobacter sp.	99.09%	KY689938.1
C2-17	1285	*Klebsiella*	*Klebsiella variicola*	97.67%	LR130544.1
			Klebsiella pneumoniae	97.67%	LR890587.1
			Klebsiella sp.	97.67%	KR189677.1
C2-18	1161	*Chryseobacterium*	*Chryseobacterium* sp.	99.66%	OK560051.1
			Chryseobacterium indologenes	99.31%	HQ647283.1
			Chryseobacterium cucumeris	99.14%	MK212371.1
C2-20	1302	*Enterobacter / Erwinia / Leclercia*	*Enterobacter* sp.	98.00%	MT321481.1
			Erwinia sp.	97.93%	LC484724.1
			Leclercia adecarboxylata	97.93%	CP040889.1
C3-22	1356	*Enterobacter / Pantoea*	*Enterobacter* sp.	98.38%	MN540105.1
			Pantoea sp.	98.08%	MK534102.1
C3-23	1162	*Leuconostoc*	*Leuconostoc holzapfelii*	98.97%	LC519862.1
			Leuconostoc citreum	98.97%	MT573003.1
			Leuconostoc holzapfelii	98.97%	MT573002.1
C3-25	1392	*Exiguobacterium*	*Exiguobacterium indicum*	99.14%	MK760069.1
			Exiguobacterium sp.	99.14%	MK415037.1

			Exiguobacterium acetylicum	99.14%	CP082333.1
C3-26	1171	*Exiguobacterium*	*Exiguobacterium indicum*	99.66%	MT214231.1
			Exiguobacterium sp.	99.14%	MK415037.1
			Exiguobacterium acetylicum	99.14%	CP082333.1
C3-27	1331	*Pseudomonas*	*Pseudomonas* sp.	99.40%	MN719064.1
			Pseudomonas monteilii	99.40%	JX144959.1
			Pseudomonas putida	99.40%	JN679861.1
C3-28	1230	*Kluyvera*	*Kluyvera ascorbata*	98.13%	FN813248.1
			Kluyvera cryocrescens	97.80%	KC686601.1
			Kluyvera sichuanensis	97.72%	MT437278.1
C4-29	1295	*Pantoea dispersa*	*Pantoea dispersa*	98.76%	MT646430.1
			Pantoea sp.	98.76%	OK563723.1
C4-30	1352	*Exiguobacterium*	*Exiguobacterium* sp.	98.96%	MK415037.1
			Exiguobacterium indicum	98.96%	MT214231.1
			Exiguobacterium acetylicum	98.96%	CP082333.1
C4-31	1390	*Klebsiella*	*Klebsiella variicola*	98.78%	CP026013.1
			Klebsiella pneumoniae	98.78%	MF682956.1
C4-32	1220	*Enterobacter / Leclercia*	*Enterobacter cloacae*	98.69%	KY400214.1
			Leclercia adecarboxylata	98.61%	CP036199.1
C5-33	1321	*Raoultella / Klebsiella*	*Raoultella* sp.	97.35%	MK600538.1
			Klebsiella aerogenes	97.35%	MK788132.1
			Raoultella ornithinolytica	97.35%	MT568560.1
			Raoultella planticola	97.35%	MT197285.1
C5-34	1431	*Exiguobacterium*	*Exiguobacterium indicum*	98.12%	MK760069.1
			Exiguobacterium acetylicum	98.12%	CP082333.1
